# An Enantiomer of an Oral Small-Molecule TSH Receptor Agonist Exhibits Improved Pharmacologic Properties

**DOI:** 10.3389/fendo.2016.00105

**Published:** 2016-07-27

**Authors:** Susanne Neumann, Umesh Padia, Mary Jane Cullen, Elena Eliseeva, Eshel A. Nir, Robert F. Place, Sarah J. Morgan, Marvin C. Gershengorn

**Affiliations:** ^1^Laboratory of Endocrinology and Receptor Biology, National Institute of Diabetes and Digestive and Kidney Diseases, National Institutes of Health, Bethesda, MD, USA

**Keywords:** TSH receptor, agonist, allosteric, enantiomer, racemic

## Abstract

We are developing an orally available small-molecule, allosteric TSH receptor (TSHR) agonist for follow-up diagnostics of patients with thyroid cancer. The agonist C2 (NCGC00161870) that we have studied so far is a racemic mixture containing equal amounts of two enantiomers, E1 and E2. As enantiomers of many drugs exhibit different pharmacologic properties, we assessed the properties of E1 and E2. We separated the two enantiomers by chiral chromatography and determined E2 as the (*S*)-(+) isomer via crystal structure analysis. E1 and E2 were shown to bind differently to a homology model of the transmembrane domain of TSHR in which E2 was calculated to exhibit lower binding energy than E1 and was, therefore, predicted to be more potent than E1. In HEK293 cells expressing human TSHRs, C2, E1, and E2 were equally efficacious in stimulating cAMP production, but their potencies were different. E2 was more potent (EC_50_ = 18 nM) than C2 (EC_50_ = 46 nM), which was more potent than E1 (EC_50_ = 217 nM). In primary cultures of human thyrocytes, C2, E1, and E2 stimulated increases in thyroperoxidase mRNA of 92-, 55-, and 137-fold and in sodium–iodide symporter mRNA of 20-, 4-, and 121-fold above basal levels, respectively. In mice, C2 stimulated an increase in radioactive iodine uptake of 1.5-fold and E2 of 2.8-fold above basal level, whereas E1 did not have an effect. C2 stimulated an increase in serum T4 of 2.4-fold, E1 of 1.9-fold, and E2 of 5.6-fold above basal levels, and a 5-day oral dosing regimen of E2 increased serum T4 levels comparable to recombinant human TSH (rhTSH, Thyrogen^®^). Thus, E2 is more effective than either C2 or E1 in stimulating thyroid function and as efficacious as rhTSH *in vivo*. E2 represents the next step toward developing an oral drug for patients with thyroid cancer.

## Introduction

Recombinant human TSH (rhTSH, Thyrogen^®^), by activating the TSH receptor (TSHR), has been used to great advantage in the initial treatment and follow-up of patients with thyroid cancer, and to a more limited extent as an adjunct in the treatment of patients with nodular goiter (1–4). rhTSH is administered via intramuscular injection over two consecutive days prior to radioactive iodine uptake (RAIU) and/or thyroglobulin (TG) testing. We have been interested in developing small-molecule ligands for the TSHR (5), which is a G protein-coupled (or seven transmembrane-spanning) receptor (GPCR) (6), and have reported on a “drug-like” TSHR agonist C2 (NCGC00161870) that was identified by high-throughput screening and subsequent chemical modification of the hit compound (7). We suggest that C2 could be used in patients in place of rhTSH. Small-molecule ligands are generally much more easily employed as drugs compared to peptides or proteins. They are synthesized chemically, can be produced in large quantities, and can typically be given orally as they are generally stable within and can be absorbed through the gastrointestinal tract. Therefore, a small-molecule TSHR agonist would offer benefits over the current standard of care to both clinicians and patients by providing a non-invasive route of drug administration at reduced economic costs as a result of patients being able to self-administer an oral bio-equivalent drug rather than requiring the need of a trained clinical professional to administer intramuscular injections of rhTSH.

C2 is a low-molecular-weight (524 Da) organic compound and was shown to bind within the transmembrane-spanning domain of TSHR and is termed an allosteric ligand since the cognate ligand TSH binds to the extracellular domain of the receptor (7). In an *in vitro* system of human thyrocytes in primary culture, C2, like TSH, induced upregulation of mRNAs for TG, thyroperoxidase (TPO), sodium–iodide symporter (NIS) and type II iodothyronine deiodinase (DIO2). *In vivo*, C2 increased serum thyroxine (T4) and thyroidal radioiodine uptake in mice (7).

C2 contains a chiral center and the racemic mixture contains two enantiomers, E1 and E2. Our studies until the present were performed with the racemic mixture containing equal amounts of the two enantiomers.

Enantiomers are two stereoisomers that are mirror images of each other and are not superimposable. The different enantiomers of a number of drugs have been shown to exhibit marked differences in biological activities, such as pharmacodynamic and pharmacokinetic properties, metabolism, and toxicology (8–10). Of particular note is that specific differences in affinity for binding to GPCRs have been found for drug enantiomers (11–13). In 1992, the United States Food and Drug Administration (FDA) issued guidelines and policies with regard to compounds that have stereochemistry (http://www.fda.gov/Drugs/GuidanceComplianceRegulatoryInformation/Guidances/ucm122883.htm). These guidelines suggest considering stereochemistry as early as possible in the search for new drug candidates, and analysis and characterization of enantiomers of a drug in early animal testing and in clinical trials. Single enantiomers have a more selective pharmacodynamics profile as compared to the racemic mixture and have lesser adverse drug reactions. Therefore, separation of enantiomers may allow for safer and more effective drugs, and may lead to drugs with improved therapeutic activity (14, 15).

Herein, we report the separation of the two enantiomers of C2 by chiral chromatography and evaluate differences in their biological effects using *in vitro* and *in vivo* systems. Our data reveal E2 is the (*S*)-(+) enantiomer, which is more effective than either C2 or the E1 enantiomer in stimulating thyroid function and fundamentally equivalent to rhTSH in our *in vivo* mouse model.

## Materials and Methods

### Chiral Separation of C2

C2 was synthesized as described previously (7). The enantiomers E1 and E2 in the racemic C2 mixture were separated by chiral chromatography using a Chiralpak IA column on a Shimadzu LC-20AT instrument (performed by Pharmaron^®^ Beijing Company, Ltd, China). The retention times of E1 and E2 were 3.64 and 4.51 min, respectively.

### Crystal Structure Determination of Enantiomer E2

The single-crystal X-ray diffraction studies were performed by Dr. Curtis Moore at the University of California, Crystallography Laboratory, San Diego, CA, USA. They were carried out on a Bruker Kappa APEX-II CCD diffractometer equipped with Cu K_α_ radiation (λ = 1.5478). Crystals of the subject compound were grown by vapor diffusion of pentane into a dichloroethane solution. A 0.153 mm × 0.055 mm × 0.031 mm colorless needle was mounted on a Cryoloop with Paratone oil. Data were collected in a nitrogen gas stream at 100(2) K using φ and ω scans. Crystal-to-detector distance was 45 mm using variable exposure time (5–30 s) depending on θ with a scan width of 1.0°. Data collection was 99.5% complete to 68.00° in θ. A total of 32,360 reflections were collected covering the indices −11 ⇐ h ⇐ 11, −13 ⇐ k ⇐ 12, −27 ⇐ l ⇐ 27. A total of 13,810 reflections were found to be symmetry independent, with a Rint of 0.0278. Indexing and unit cell refinement indicated a primitive, triclinic lattice. The space group was found to be *P*1. The data were integrated using the Bruker SAINT Software program (Bruker AXS Inc., Madison, WI, USA) and scaled using the SADABS Software program. Solution by direct methods (SHELXT) (16) produced a complete phasing model consistent with the proposed structure. All non-hydrogen atoms were refined anisotropically by full-matrix least-squares (SHELXL-2014). All hydrogen atoms were placed using a riding model. Their positions were constrained relative to their parent atom using the appropriate HFIX command in SHELXL-2014. Crystallographic data are available upon request.

### Molecular Modeling of TSHR

A homology model was generated of the transmembrane region of TSHR. A BLAST (Basic Local Alignment Search Tool) search (17) was conducted to find a template receptor with a solved structure that had high sequence identity to TSHR. Due to high sequence identity and hypothesized similarities between active conformations of both receptors, the crystallographic structure of active bovine rhodopsin (PDB: 2X72) at 2.8Å resolution was ultimately chosen. The alignment between the sequences of these receptors was initially achieved using ClustalX version 2.1 (18), with refinement conducted manually for the looped regions.

The model of TSHR was determined through the combined information contained within the alignment and the crystallographic structure of the template receptor using the library MODELLER version 9.15 (19, 20). Based on these initial conditions, 50 potential models were generated and ranked by objective scoring functions (DOPE, GA341). A final model was selected based on favorable scores and conserved geometry determined by PROCHECK (21). Molecular dynamics (MD) simulations were used to refine the model of TSHR by modeling the receptor in a similar setting to that of the receptor *in vivo*. The receptor was embedded into a 1,2-dilauroylphosphatidic acid lipid bilayer and solvated by water and potassium or chloride ions. The final MD simulation was carried out using the GROMACS version 5.0 MD package (22). The calculations were performed with the CHARMM (Chemistry at Harvard Molecular Mechanics) force field (23) for 100 ns at 310°K. The simulation was run on Biowulf2, a massively parallelized high performance cluster at the National Institutes of Health (NIH). A representative model was chosen from these simulations for docking studies. Autodock Vina (24) was used to dock E1 and E2.

### Measurement of cAMP Production

HEK293 cells stably expressing TSHR (HEK-TSHR), luteinizing hormone receptor (LHR) (HEK-LHR), or follicle-stimulating hormone receptor (FSHR) (HEK-FSHR) were described previously (25). Cells were grown in Dulbecco’s modified Eagle’s medium (DMEM) supplemented with 10% fetal bovine serum, 100 units/ml penicillin and 10 μg/ml streptomycin (Life Technologies Corporation, Carlsbad, CA, USA) at 37°C in a humidified 5% CO_2_ incubator. Twenty-four hours before the experiment, 1.1 × 10^5^ cells/ml were seeded in 48-well plates. Subsequently, growth medium was replaced by HBSS/10 mM HEPES containing 1 mM 3-isobutyl-1-methylxanthine (IBMX) (Sigma-Aldrich, St. Louis, MO, USA) and cells were incubated with small-molecule ligands C2, E1, or E2 in a humidified 5% CO_2_ incubator at 37^o^C for 1 h. Following aspiration of the medium, cells were lysed using lysis buffer of the cAMP-Screen Direct^TM^ System (Life Technologies Corporation). The cAMP content of the cell lysate was determined using the method described in the manufacturer’s protocol. The potencies (i.e. EC_50_s) of the compounds were obtained from the dose–response curves using GraphPad Prism Version 5 for Windows (GraphPad Software, La Jolla, CA, USA).

### Primary Cultures of Human Thyrocytes

Human thyrocytes were isolated from normal thyroid tissue samples from patients undergoing thyroidectomy for thyroid cancer at the National Institutes of Health Clinical Center. Specimens were obtained under National Institute of Diabetes and Digestive and Kidney Diseases (NIDDK) Institutional Review Board approved protocols after informed consent was obtained from patients. Cultures of primary thyrocytes were established as described previously (7).

### Measurement of Sodium–Iodide Symporter and Thyroperoxidase Expression in Primary Cultures of Human Thyrocytes

Thyrocytes (0.5 × 10^5^ cells/well) were seeded into 24-well plates in DMEM containing 10% FBS. Twenty-four hours prior to the experiment, media were changed to 0.1% bovine serum albumin (BSA)-containing DMEM. Cells were stimulated with C2, E1, or E2 for the indicated times.

Total RNA was purified using RNeasy Mini Kits (Qiagen Inc., Valencia, CA, USA). First-strand cDNA was prepared using a High Capacity cDNA Reverse Transcription Kit (Life Technologies Corporation). RT-PCR was performed in 25 μl reactions using cDNA prepared from 100 ng or less of total RNA and TaqMan Universal PCR Master Mix (Life Technologies Corporation). Levels of mRNA were measured using primers and probes from Life Technologies Corporation. Quantitative RT-PCR results were normalized to GAPDH to correct for differences in RNA input.

### Measurement of Serum Total T4 in Mice

All animals were maintained at NIH facilities in accordance with the guidelines established by the Association for Assessment and Accreditation of Laboratory Animal Care (AALAC) and all protocols were approved by the Institutional Animal Care and Use Committee at the NIDDK. Mice were housed up to five per cage and maintained on a 12:12 h light–dark cycle with lights out at 06:00 p.m. The animal room was maintained at 22°C and 50% humidity. Mice were maintained on standard rodent chow (NIH-31; Zeigler Brothers, Inc., Gardners, PA, USA). Water and feed were available *ad libitum*. At the time of experiments, mouse ages ranged from 8 to 13 weeks.

#### Comparison of C2, E1, and E2 regarding the Increase of Serum Total T4

Experiments to compare the racemic mixture C2 with the enantiomers E1 and E2 were carried out in female BALB/c mice (National Cancer Institute Animal Production Program, Fredrick, MD, USA). Compounds were dissolved in a vehicle consisting of 100% polyethylene glycol 300 (PEG 300) (Hampton Research, Aliso Viejo, CA, USA). 3,3′,5-Triiodo-l-thyronine sodium (T3, Sigma-Aldrich) was dissolved in 0.1 N sodium hydroxide (NaOH) (Sigma-Aldrich). T3 was further diluted to a concentration of 50 μg/ml with phosphate buffered saline (PBS).

The treatment groups were vehicle, C2, E1, and E2 at 0.5 or 1 mg per animal. Animals were dosed via intraperitoneal injection. T3 (5 μg/animal) was given intraperitoneally in the morning of each treatment day to inhibit endogenous TSH secretion. C2, E1, or E2 were given once or twice daily. Serum was obtained by terminal retro-orbital bleed from anesthetized mice 4 h after the last injection.

#### Optimization of E2 Dosing Schedule, and Comparison of E2 and rhTSH regarding the Increase of Serum Total T4

Experiments to optimize the dosing schedule of E2, and to compare E2 to rhTSH were performed in female CD1 mice (Charles River Laboratories, Bar Harbor, ME, USA). E2 and T3 were dissolved as described above. T3 was further diluted to a concentration of 3 μg/ml with distilled water (T3 water). rhTSH (Thyrogen^®^) was purchased from Sanofi Genzyme, Cambridge, MA, USA. The drinking water was replaced with T3 water 6 days prior to treatment to inhibit endogenous TSH secretion. Two milligram E2 per animal was administered orally once or twice a day for two or five consecutive days. Four microgram rhTSH per animal (a converted human equivalent dose) was given intraperitoneally once a day on two consecutive days. Serum was obtained by terminal retro-orbital bleed from anesthetized mice 2 h after the last injection. Total T4 levels were measured with the GammaCoat^TM^ T4 Radioimmunoassay (DiaSorin Inc., Stillwater, MN, USA) using the manufacturer’s protocol.

### Thyroidal Radioiodine Uptake in Mice

Experiments were performed with female CD1 mice. At the time of experiments, mouse ages ranged from 8 to 10 weeks. C2, E1, and E2 were dissolved in a vehicle consisting of 10% *N,N*-dimethylacetamide (Sigma Aldrich) and 90% PEG 300 (Hampton Research). Na^125^I (Perkin-Elmer, Waltham, MA, USA) with a specific activity of 17 Ci/mg in 0.1 N NaOH was diluted in PBS to provide an activity of 100 μCi/ml.

To measure iodine uptake, animals were assigned to one of four treatment groups based on body weight. Their drinking water was replaced with T3 water 6 days prior to treatment to inhibit endogenous TSH secretion. They were subsequently maintained on the T3 water until the conclusion of the experiment. The treatment groups were Control (vehicle), C2, E1, and E2 at 2 or 10 mg per animal. Animals were dosed via oral gavage with either vehicle or test compound once a day for 2 days. One the third day, they were injected intraperitoneally with 200 μl (20 μCi) of ^125^I. Twenty-four hours later their thyroid glands and livers (as a control) were excised and counted in a gamma counter (Perkin Elmer Model 1470, Perkin Elmer). Iodine uptake was measured as the number of counts per minute.

### Statistical Analysis

Statistical analysis was performed by GraphPad Prism version 5 for Windows. Data are presented as mean ± SE values. Statistically significant differences among groups were assessed by *t*-test.

## Results and Discussion

**Figure 1:**
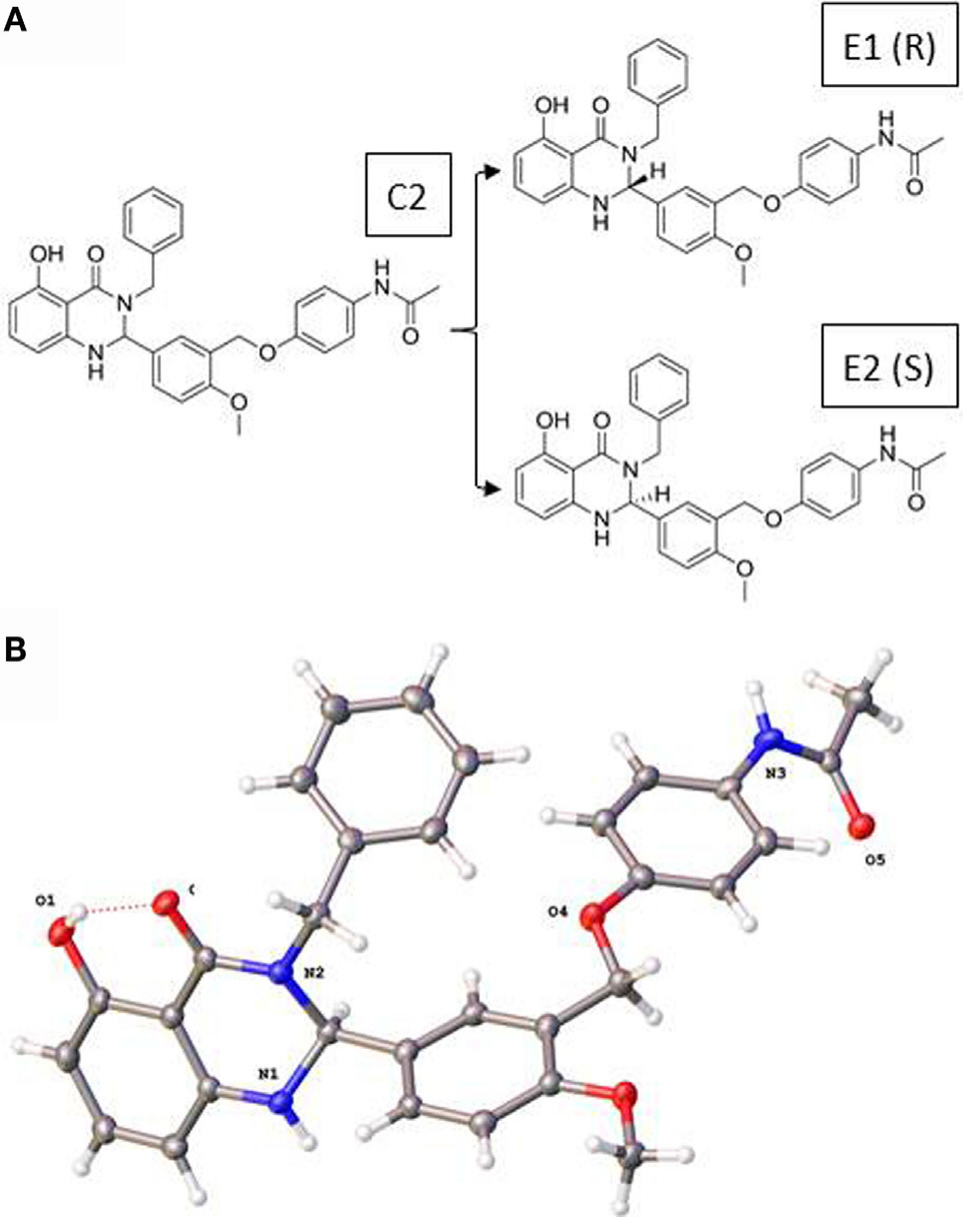
**(A)** Two-dimensional structures of the racemic mixture C2 and of the enantiomers E1 and E2. **(B)** Crystal structure of the (S)-(+)-enantiomer E2.

**Figure 2:**
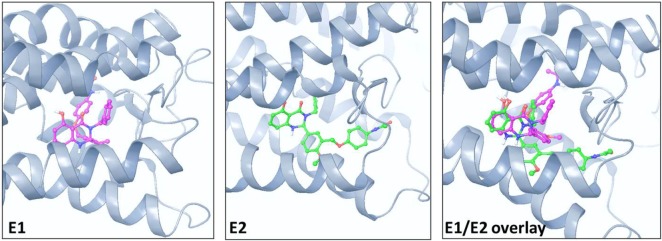
**Comparison of the poses and spatial locations of E1 (magenta) and E2 (green) in the homology model of TSHR**. Both compounds docked into the allosteric binding site within the transmembrane serpentine domain of TSHR. The E1/E2 overlay shows that the orientations and TSHR interacting amino acid residues are different for E1 and E2.

Figure 1 illustrates the 2D structures of the racemic mixture C2, the enantiomers E1 and E2 (A), and the crystal structure of E2 (B). The crystal structure of E2 shows it to be the (*S*)-(+)-enantiomer. Figure 2 shows comparisons of the docking of E1 and E2 to the serpentine transmembrane domain of the homology model of TSHR. We demonstrated previously (7) that the racemic mixture C2 binds in this region with specific interactions with an asparagine residue (N590) in transmembrane helix 5 [N5.47 in the Ballesteros and Weinstein nomenclature (26)]. This new TSHR model confirms the previous binding pocket and shows important differences in the binding of E1 and E2. The resulting conformations of both enantiomers were situated in the transmembrane domain of TSHR. However, the binding energy of E2 was significantly more favorable (−9.2 kcal/mol) than that of E1 (−5.6 kcal/mol). Upon further evaluation of the interactions between each enantiomer and the receptor, E2 had several potential hydrogen bonding and π–π interactions with key amino acid residues [T501 (T3.32), V502 (V3.33), S505 (S3.36), E506 (3.37), F585 (F5.42), S641 (S6.52), K660 (K7.35), Y667 (Y7.42)], while E1 generally had weaker lipophilic and hydrophilic interactions, along with just two π–π interactions. Thus, E2 is predicted to be a more potent TSHR agonist than E1 based on the enantiomer binding model.

**Figure 3:**
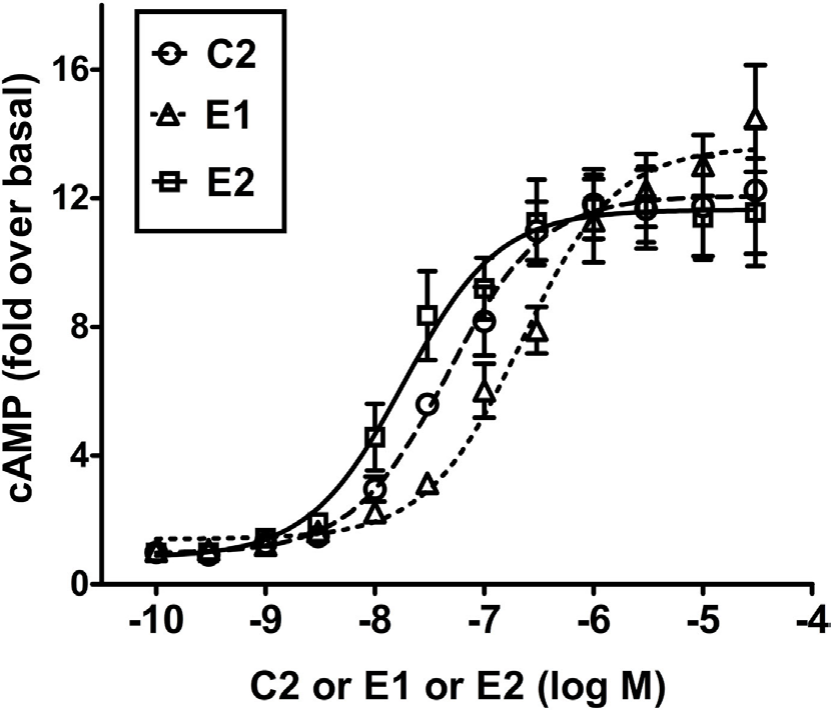
**E2 is more potent than C2 that is more potent than E1 in stimulating cAMP production in HEK-TSHR cells**. Cells were stimulated by increasing doses (0–30 μM) of C2, E1, or E2 as described in Section “Materials and Methods.” The intracellular cAMP levels were measured after 1 h incubation with the small-molecule ligands by ELISA. The data are from three experiments with duplicate samples and are presented as mean ± SE.

Figure 3 illustrates a comparison of the dose-dependent stimulation of cAMP production in HEK-TSHR cells. We use these engineered cells because they exhibit a robust cAMP signal and allow differences in potencies of different agonists to be quantified. Like C2 (7), E1 and E2 stimulated cAMP production in cells expressing TSHRs but not in cells expressing LHRs or FSHRs, which have high homology to TSHR in the transmembrane domain (data not shown). Thus, E1 and E2 are specific agonists for TSHR. C2, E1, and E2 exhibited similar maximal increases in cAMP production but different potencies. E2 was more potent (EC_50_ = 18 nM) than C2 (EC_50_ = 46 nM), which was more potent than E1 (EC_50_ = 217 nM). These findings supported the predictions from the homology models and predicted that E2 would be more active than C2 and E1 in other assays of thyroid cell function.

**Figure 4:**
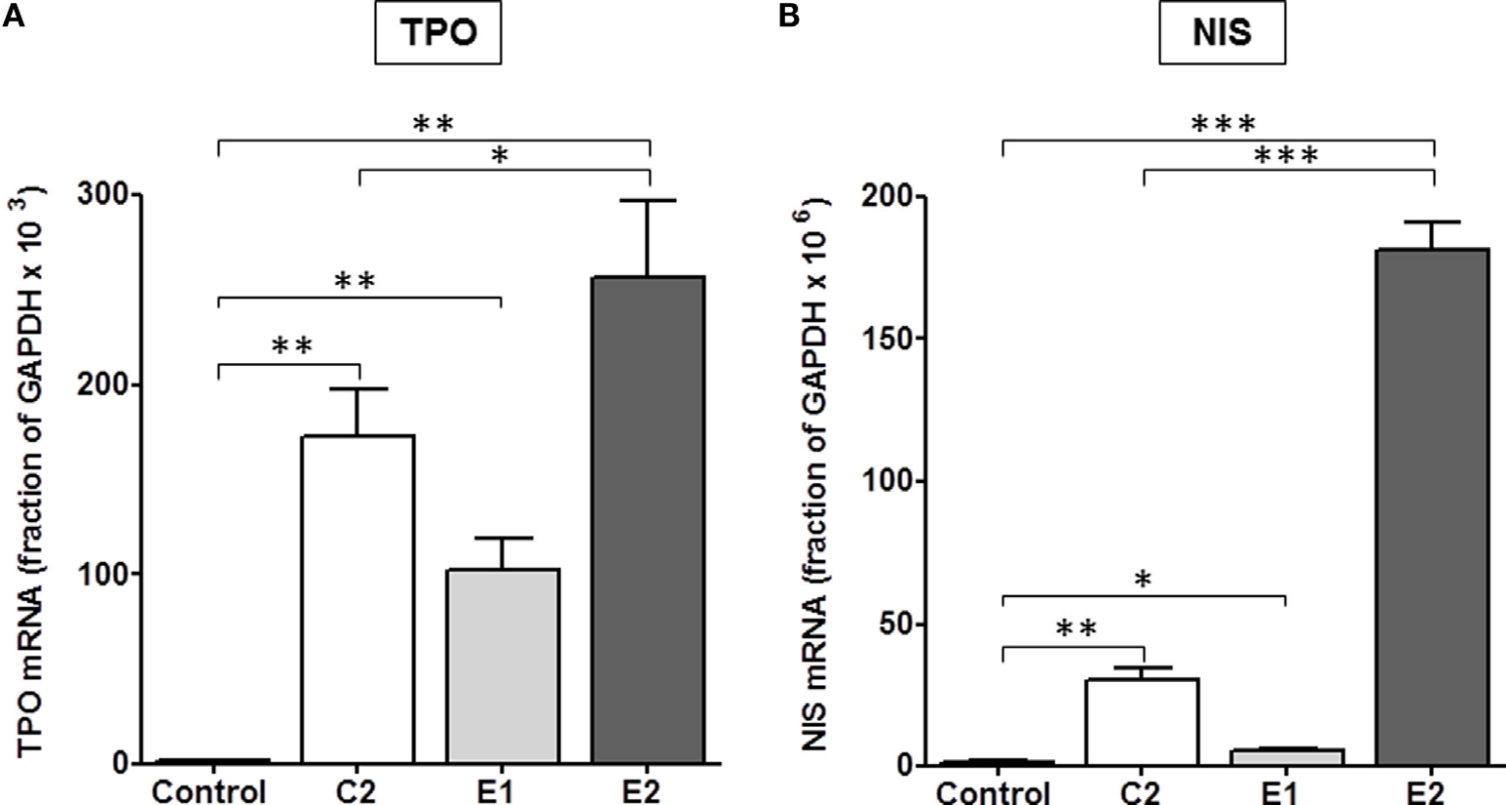
**E2 was more effective than C2 that was more effective than E1 in upregulating the gene expression of TPO (A) and NIS (B) in primary cultures of human thyrocytes**. Cells were stimulated with 10 μM C2, E1, or E2 for 5 days. mRNA levels were measured by quantitative RT-PCR. The data represent the mean ± SE in two experiments with duplicate samples. Data were analyzed by *t*-test; TPO: **p < 0.01, C2, E1, and E2 versus Control; *p < 0.1, E2 versus C2; NIS: ***p < 0.001, E2 versus Control, and E2 versus C2; **p < 0.01, C2 versus Control; *p < 0.1, E1 versus Control.

It was important to confirm this hierarchy in signaling in a more physiologically relevant system with endogenously expressed TSHRs. We used primary cultures of human thyrocytes and measured the effects of all three TSHR agonists on upregulation of thyroid-specific mRNA levels of NIS and TPO. Thyroidal iodide influx, iodide efflux, and iodide organification are mediated by NIS (27–29) and determine radioactive iodine accumulation. TSH is the main regulator of NIS transcription in normal thyroid cells (30) and increases NIS mRNA and protein levels (31, 32). TPO plays a key role in thyroid hormone synthesis by catalyzing both the iodination of tyrosine residues and the coupling of iodotyrosine residues in TG, resulting in the formation of T3 and T4 (33). Figure 4 illustrates the effects of C2, E1, and E2 on upregulation of TPO and NIS gene expression. A time course over 8 days showed a maximum increase in NIS and TPO gene expression on day 5 (data not shown). In cells stimulated by 10 μM doses of each compound for 5 days, C2 stimulated an increase in thyroidal NIS mRNA of 20 ± 2.5-fold, E1 of 3.6 ± 0.6-fold, and E2 of 121 ± 6.4-fold above basal levels. Similarly, TPO mRNA increased over basal 92 ± 13-fold for C2, 55 ± 9-fold for E1, and 137 ± 21-fold for E2. Thus, E2 was more effective in upregulating NIS and TPO gene expression than either C2 or the E1 enantiomer. These findings in primary cultures of human thyrocytes are consistent with those found in the engineered HEK-TSHR cells (Figure 3).

**Figure 5:**
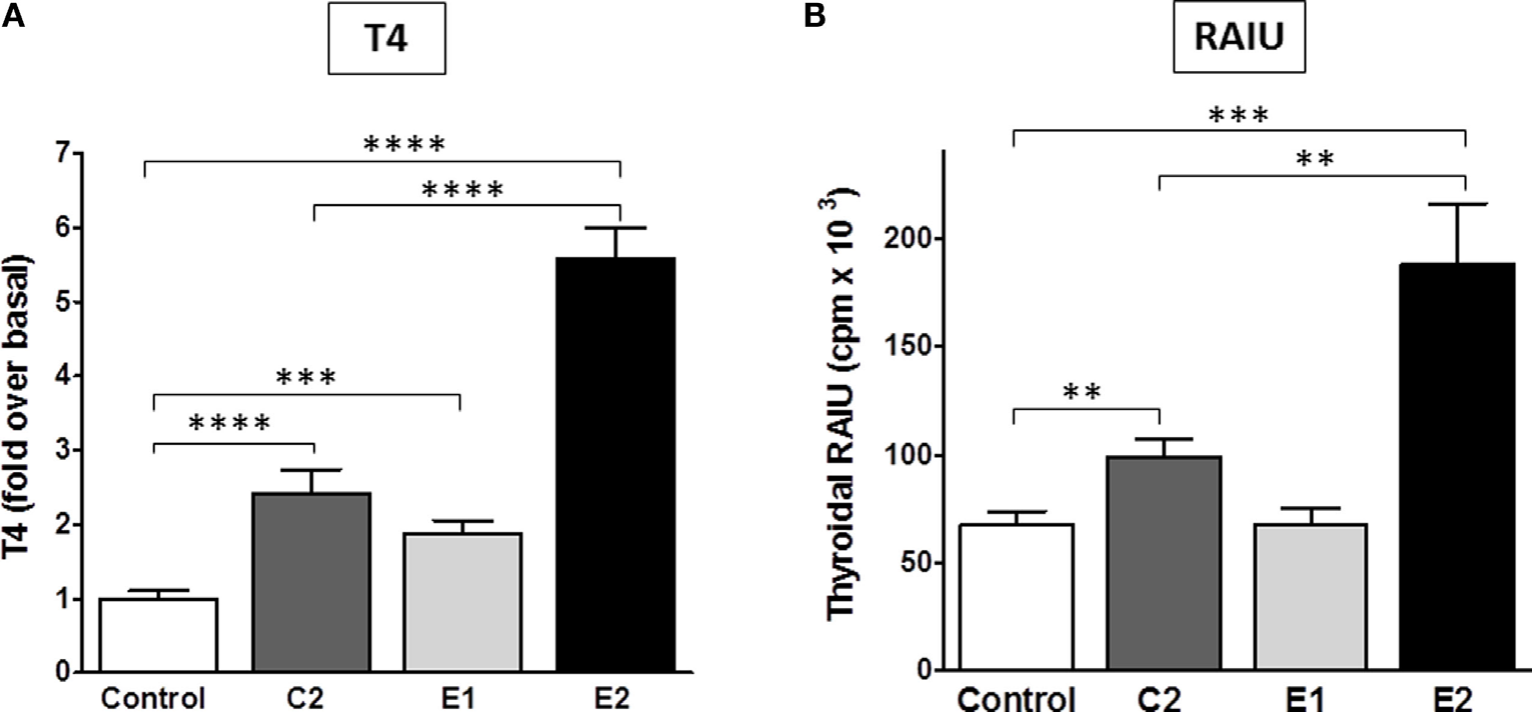
**Stimulation of T4 secretion and thyroidal radioiodine uptake in mice by C2, E1, and E2**. **(A)** Serum total T4 was measured in three independent experiments. The sum of all data is presented as mean ± SE. The individual experiments are shown in Figure S1 in Supplementary Material. The experiments had four to six mice per treatment group. T3 (5 μg/mouse) was given intraperitoneally in the morning of each treatment day to inhibit endogenous TSH secretion. Animals were dosed with TSHR ligands via intraperitoneal injection. Experiment 1: 0.5 mg of each compound was given in the afternoon of day 1, in the morning and afternoon of day 2, and in the morning of day 3. Experiments 2 and 3: 0.5 or 1 mg of each compound, respectively, was given twice a day on days 1 and 2, and one morning dose was given on day 3. Serum was obtained by terminal retro-orbital bleed from anesthetized mice 4 h after the last injection. Data were analyzed by *t*-test; ****p < 0.0001, C2, and E2 versus Control, E2 versus C2; ***p < 0.001, E1 versus Control. There is no significant difference between C2 and E1. **(B)** Radioiodide uptake: animals were dosed via oral gavage with either vehicle or test compound (2 mg in experiment 1 and 10 mg in experiment 2) once a day for 2 days. Each experiment had five mice per treatment group (total *n* = 10). On the third day, mice were injected intraperitoneally with Na^125^I. Twenty-four hours later, radioiodine uptake was measured. The results of two experiments were combined and are presented as mean ± SE. The data were analyzed by *t*-test; ***p < 0.001, E2 versus Control; **p < 0.01, C2 versus Control, and E2 versus C2. There is no significant difference between Control and E1.

Most importantly, we determined whether there were differences between the enantiomers in stimulating biologic responses in mice. Figure 5 shows the effects of C2, E1, and E2 on two measurements of thyroid function: (i) T4 secretion in serum, and (ii) RAIU. For T4 levels, the same hierarchy was found – E2 > C2 > E1 – as was revealed in the *in vitro* studies. Figure 5A summarizes the results of three independent experiments. In mice administered 0.5 mg (experiments 1 and 2) or 1 mg (experiment 3) of each compound intraperitoneally twice a day, C2 stimulated an increase in serum T4 of 2.4 ± 0.3-fold, E1 of 1.9 ± 0.2-fold, and E2 of 5.7 ± 0.4-fold above basal levels. The data of each individual experiment are presented in Figure S1 in Supplementary Material.

Radioactive iodine thyroid scans are commonly used to diagnose residual and/or recurrent thyroid cancer. For the last decade, rhTSH has been used in the follow-up of thyroidectomy to increase the sensitivity for detection of recurrent or metastatic thyroid cancer in patients who undergo regular monitoring (2). In addition, rhTSH was approved by the FDA for a supplemental indication to improve radioiodine ablation of thyroid remnants after surgical thyroidectomy in patients with thyroid cancer (34). A small-molecule TSHR agonist could replace rhTSH because it could produce the same beneficial effects but with greater ease of oral administration and, therefore, would be available for use in a larger patient population. Figure 5B represents a summary of two independent RAIU experiments with five animals per condition in each experiment (total *n* = 10). The RAIU in mice showed the same order of enantiomer activity as observed in the serum T4 assay. E1 did not increase RAIU whereas E2 was significantly more effective than C2. Two or 10 mg of each agonist were given orally once daily on two consecutive days in the first and second experiment, respectively. C2 stimulated an increase in RAIU of 1.5 ± 0.1-fold, and E2 of 2.8 ± 0.4-fold above basal levels.

**Figure 6:**
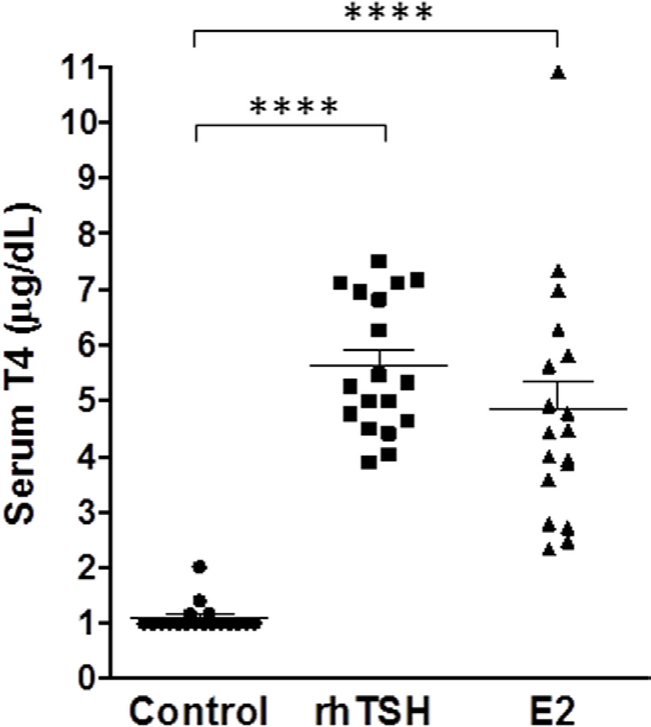
**E2 increases serum T4 levels comparable to rhTSH**. The drinking water was replaced with T3 water 6 days prior to treatment. Two milligram E2 per animal was administered orally twice a day for five consecutive days or 4 μg rhTSH per animal was given intraperitoneally once a day on two consecutive days. Serum for T4 measurement was obtained by terminal retro-orbital bleed from anesthetized mice 2 h after the last dose. The results of two experiments ± SE are presented. The total number of animals per treatment group was 18. Data were analyzed by *t*-test; ****p < 0.0001, E2, and rhTSH versus Control.

In clinical practice, rhTSH is administered intramuscularly on two consecutive days. Our *in vitro* data in primary cultures of human thyrocytes demonstrated that upregulation of NIS gene expression by E2 reached a maximum on day 5. Therefore, we optimized our dosing schedule and frequency to maximize the E2 response *in vivo*. We measured serum T4 levels to further evaluate E2 efficacy *in vivo* since it was the primary readout used to validate the pre-clinical efficacy of rhTSH within its Investigational New Drug (IND) application for the FDA. Mice were given free access to T3 water for 6 days before the treatment with E2 or rhTSH to suppress endogenous TSH levels. In an initial experiment, CD1 mice (*n* = 4 per treatment condition) were dosed with 2 mg E2 orally once a day for 2 or 5 days. rhTSH was administered via intraperitoneal injection on two consecutive days at a converted human equivalent dose equal to 4 μg/animal/day. As shown in Figure S2 in Supplementary Material, vehicle treated animals had T4 levels of 1.3 ± 0.2 μg/dl. E2 treatment for 2 days led to an increase in total T4 over basal (2.0 ± 0.3 μg/dl). There was a further increase in total T4 with E2 treatment for 5 days (3.5 ± 0.5 μg/dl) and, importantly, no significant difference when compared to the effect induced by rhTSH (4.2 ± 0.5 μg/dl). In two subsequent experiments (total *n* = 18 per treatment group) (Figure 6), 2 mg E2 per animal was given orally twice a day on five consecutive days and 4 μg rhTSH per animal was given via intraperitoneal injection once a day on two consecutive days. Vehicle treated mice had a total T4 of 1.1 ± 0.1 μg/dl. E2 and rhTSH increased serum T4 levels to 4.8 ± 0.5 μg/dl and 5.6 ± 0.3 μg/dl, respectively, with no significant difference between E2 and rhTSH.

In summary, we found that separating the two enantiomers of the racemic mixture C2 has allowed for generation of a novel molecular enantiomer E2 that is more potent in stimulating TSHR effects *in vitro* and more active in stimulating thyroid function *in vivo* in mice than C2 or E1. Oral administration of E2 is just as efficacious as intraperitoneal injections of rhTSH in our *in vivo* mouse model. This is an important step in the pre-clinical development of a small-molecule TSHR agonist to stimulate radioiodine uptake and/or serum TG levels in patients with thyroid cancer and supports, in part, proceeding to first-in-human trials.

## Author Contributions

All authors have contributed significantly to the work, have read the manuscript, attest to the validity and legitimacy of the data and its interpretation, and agree to its submission.

## Conflict of Interest Statement

SN and MG are co-inventors on a patent for these compounds. The authors declare that the research was conducted in the absence of any commercial or financial relationships that could be construed as a potential conflict of interest. The reviewer SF and handling Editor declared their shared affiliation, and the handling Editor states that the process nevertheless met the standards of a fair and objective review.

## References

[1] Bonnema SJ Fast S Hegedus L. The role of radioiodine therapy in benign nodular goitre. Best Pract Res Clin Endocrinol Metab (2014) 28(4):619–31. 10.1016/j.beem.2014.02.001 25047210

[2] Duntas LH Cooper DS. Review on the occasion of a decade of recombinant human TSH: prospects and novel uses. Thyroid (2008) 18(5):509–16. 10.1089/thy.2007.0331 18426363

[3] Woodmansee WW Haugen BR. Uses for recombinant human TSH in patients with thyroid cancer and nodular goiter. Clin Endocrinol (Oxf) (2004) 61(2):163–73. 10.1111/j.1365-2265.2004.02025.x 15272910

[4] Klubo-Gwiezdzinska J Burman KD Van ND Mete M Jonklaas J Wartofsky L. Potential use of recombinant human thyrotropin in the treatment of distant metastases in patients with differentiated thyroid cancer. Endocr Pract (2013) 19(1):139–48. 10.4158/EP12244.RA 23186979 PMC4185285

[5] Gershengorn MC Neumann S. Update in TSH receptor agonists and antagonists. J Clin Endocrinol Metab (2012) 97(12):4287–92. 10.1210/jc.2012-3080 23019348 PMC3513532

[6] Kleinau G Hoyer I Kreuchwig A Haas AK Rutz C Furkert J From molecular details of the interplay between transmembrane helices of the thyrotropin receptor to general aspects of signal transduction in family a G-protein-coupled receptors (GPCRs). J Biol Chem (2011) 286(29):25859–71. 10.1074/jbc.M110.196980 21586576 PMC3138303

[7] Neumann S Huang W Titus S Krause G Kleinau G Alberobello AT Small-molecule agonists for the thyrotropin receptor stimulate thyroid function in human thyrocytes and mice. Proc Natl Acad Sci USA (2009) 106(30):12471–6. 10.1073/pnas.0904506106 19592511 PMC2708975

[8] Smith SW. Chiral toxicology: it’s the same thing…only different. Toxicol Sci (2009) 110(1):4–30. 10.1093/toxsci/kfp097 19414517

[9] Williams K Lee E Importance of drug enatiomers in clinical pharmacology. Drugs (1985) 30(4):333–54. 10.2165/00003495-198530040-00003 3905334

[10] Nguyen LA He H Pham-Huy C. Chiral drugs: an overview. Int J Biomed Sci (2006) 2(2):85–100. 23674971 PMC3614593

[11] Kieffer BL Befort K Gaveriaux-Ruff C Hirth CG The delta-opioid receptor: isolation of a cDNA by expression cloning and pharmacological characterization. Proc Natl Acad Sci USA (1992) 89(24):12048–52. 10.1073/pnas.89.24.12048 1334555 PMC50695

[12] Nemeth EF Steffey ME Hammerland LG Hung BC Van Wagenen BC DelMar EG Calcimimetics with potent and selective activity on the parathyroid calcium receptor. Proc Natl Acad Sci U S A (1998) 95(7):4040–5. 10.1073/pnas.95.7.4040 9520489 PMC19959

[13] Pertwee RG. Pharmacology of cannabinoid CB1 and CB2 receptors. Pharmacol Ther (1997) 74(2):129–80. 10.1016/S0163-7258(96)00204-5 9336020

[14] Brooks WH Guida WC Daniel KG. The significance of chirality in drug design and development. Curr Top Med Chem (2011) 11(7):760–70. 10.2174/156802611795165098 21291399 PMC5765859

[15] Chhabra N Aseri ML Padmanabhan D A review of drug isomerism and its significance. Int J Appl Basic Med Res (2013) 3(1):16–8. 10.4103/2229-516X.112233 23776834 PMC3678675

[16] Sheldrick GM. Crystal structure refinement with SHELXL. Acta Crystallogr C Struct Chem (2015) 71(1):3–8. 10.1107/S2053229614024218 25567568 PMC4294323

[17] Altschul SF Madden TL Schaffer AA Zhang J Zhang Z Miller W Gapped BLAST and PSI-BLAST: a new generation of protein database search programs. Nucleic Acids Res (1997) 25(17):3389–402. 10.1093/nar/25.17.3389 9254694 PMC146917

[18] Thompson JD Gibson TJ Plewniak F Jeanmougin F Higgins DG. The CLUSTAL_X windows interface: flexible strategies for multiple sequence alignment aided by quality analysis tools. Nucleic Acids Res (1997) 25(24):4876–82. 10.1093/nar/25.24.4876 9396791 PMC147148

[19] Fiser A Sali A Modeller: generation and refinement of homology-based protein structure models. Methods Enzymol (2003) 374:461–91. 10.1016/S0076-6879(03)74020-8 14696385

[20] Sali A Blundell TL. Comparative protein modelling by satisfaction of spatial restraints. J Mol Biol (1993) 234(3):779–815. 10.1006/jmbi.1993.1626 8254673

[21] Laskowski RA Rullmannn JA MacArthur MW Kaptein R Thornton JM. AQUA and PROCHECK-NMR: programs for checking the quality of protein structures solved by NMR. J Biomol NMR (1996) 8(4):477–86. 10.1007/BF00228148 9008363

[22] van der Spoel D Lindahl E Hess B Groenhof G Mark AE Berendsen HJ. GROMACS: fast, flexible, and free. J Comput Chem (2005) 26(16):1701–18. 10.1002/jcc.20291 16211538

[23] Brooks BR Brooks CLIII MacKerell AD Jr Nilsson L Petrella RJ Roux B CHARMM: the biomolecular simulation program. J Comput Chem (2009) 30(10):1545–614. 10.1002/jcc.21287 19444816 PMC2810661

[24] Trott O Olson AJ. AutoDock Vina: improving the speed and accuracy of docking with a new scoring function, efficient optimization, and multithreading. J Comput Chem (2010) 31(2):455–61. 10.1002/jcc.21334 19499576 PMC3041641

[25] Neumann S Kleinau G Costanzi S Moore S Jiang JK Raaka BM A low-molecular-weight antagonist for the human thyrotropin receptor with therapeutic potential for hyperthyroidism. Endocrinology (2008) 149(12):5945–50. 10.1210/en.2008-0836 18669595 PMC2613050

[26] Ballesteros JA Weinstein H Integrated methods for the construction of three-dimensional models and computational probing of structure-function relations in G protein coupled receptors. Methods Neurosci (1995) 25:366–428. 10.1016/S1043-9471(05)80049-7

[27] Dai G Levy O Carrasco N. Cloning and characterization of the thyroid iodide transporter. Nature (1996) 379(6564):458–60. 10.1038/379458a0 8559252

[28] Kopp PA Reduce, recycle, reuse – iodotyrosine deiodinase in thyroid iodide metabolism. N Engl J Med (2008) 358(17):1856–9. 10.1056/NEJMe0802188 18434655

[29] Smanik PA Liu Q Furminger TL Ryu K Xing S Mazzaferri EL Cloning of the human sodium lodide symporter. Biochem Biophys Res Commun (1996) 226(2):339–45. 10.1006/bbrc.1996.1358 8806637

[30] Taki K Kogai T Kanamoto Y Hershman JM Brent GA. A thyroid-specific far-upstream enhancer in the human sodium/iodide symporter gene requires Pax-8 binding and cyclic adenosine 3′,5′-monophosphate response element-like sequence binding proteins for full activity and is differentially regulated in normal and thyroid cancer cells. Mol Endocrinol (2002) 16(10):2266–82. 10.1210/me.2002-0109 12351692

[31] Kogai T Endo T Saito T Miyazaki A Kawaguchi A Onaya T. Regulation by thyroid-stimulating hormone of sodium/iodide symporter gene expression and protein levels in FRTL-5 cells. Endocrinology (1997) 138(6):2227–32. 10.1210/endo.138.6.5189 9165005

[32] Saito T Endo T Kawaguchi A Ikeda M Nakazato M Kogai T Increased expression of the Na+/I- symporter in cultured human thyroid cells exposed to thyrotropin and in Graves’ thyroid tissue. J Clin Endocrinol Metab (1997) 82(10):3331–6. 10.1210/jcem.82.10.4269 9329364

[33] Ruf J Carayon P. Structural and functional aspects of thyroid peroxidase. Arch Biochem Biophys (2006) 445(2):269–77. 10.1016/j.abb.2005.06.023 16098474

[34] Lee J Yun MJ Nam KH Chung WY Soh EY Park CS. Quality of life and effectiveness comparisons of thyroxine withdrawal, triiodothyronine withdrawal, and recombinant thyroid-stimulating hormone administration for low-dose radioiodine remnant ablation of differentiated thyroid carcinoma. Thyroid (2010) 20(2):173–9. 10.1089/thy.2009.0187 20151824

