# Supplementary material for: An Enantiomer of an Oral Small-Molecule TSH Receptor Agonist Exhibits Improved Pharmacologic Properties
Source: Front Endocrinol (Lausanne). 2016 Jul 27;7:105. doi: 10.3389/fendo.2016.00105 (PMC4961696; doi:10.3389/fendo.2016.00105)
Supplement: Supplementary file 1 [file Presentation1.PDF]

Supplementary Material

An Enantiomer of an Oral Small Molecule TSH Receptor Agonist Exhibits Improved Pharmacologic Properties

Susanne Neumann*, Umesh Padia, Mary Jane Cullen, Elena Eliseeva, Eshel A. Nir, Robert F. Place, Sarah J. Morgan, and Marvin C. Gershengorn

***Correspondence:** Susanne Neumann

Email: susannen@intra.niddk.nih.gov

Supplementary Figures

Supplementary Figure 1: Stimulation of T4 secretion by C2, E1, and E2

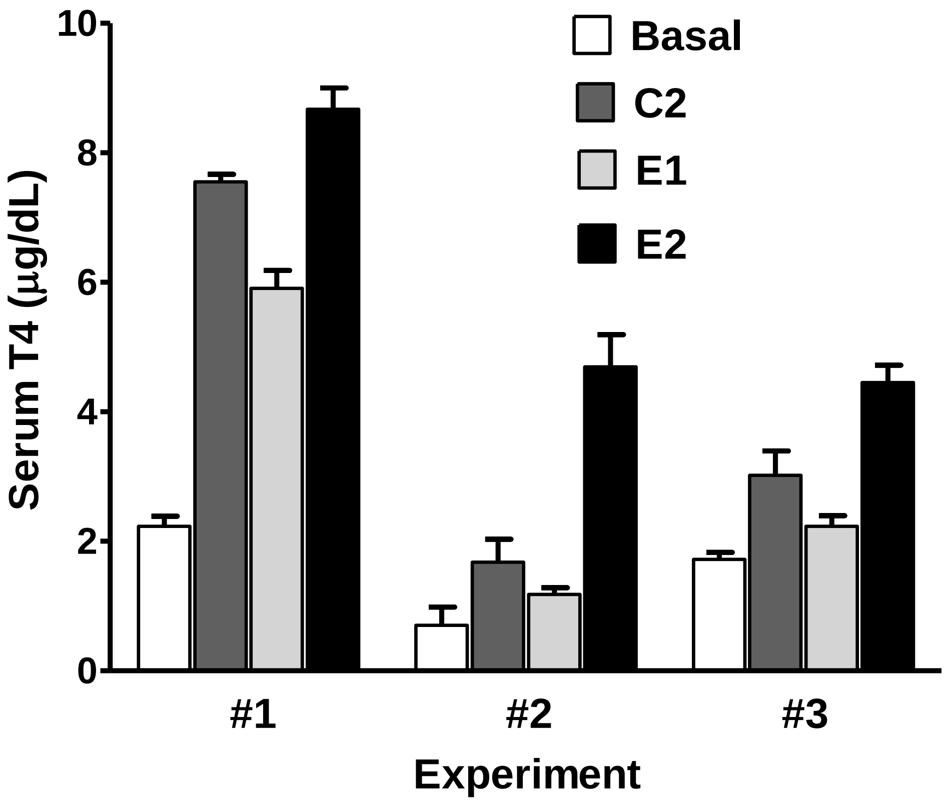

Serum total T4 was measured in 3 experiments. The experimental conditions vary between the experiments; therefore, the results of each experiment are shown. The experiments had 4 to 6 mice per treatment group. T3 (5 μg/mouse) was given intraperitoneally in the morning of each treatment day to inhibit endogenous TSH secretion. Animals were dosed with TSHR ligands via intraperitoneal injection. Experiment #1: 0.5 mg of each compound was given in the afternoon of day 1, in the morning and afternoon of day 2, and in the morning of day 3. Experiment #2 and #3: 0.5 mg or 1 mg of each compound, respectively, was given twice a day on day 1 and 2, and one morning dose was given on day 3. Serum was obtained by terminal retro-orbital bleed from anesthetized mice 4 h after the last injection.

Supplementary Figure 2: Optimization of E2 dosing schedule.

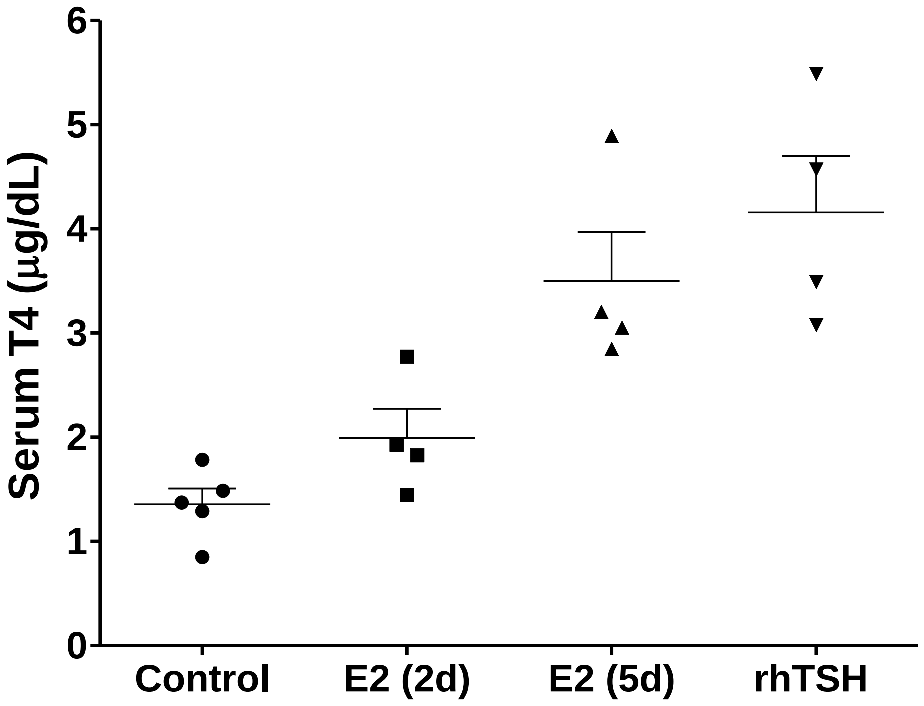

The drinking water was replaced with T3 water (3µg/ml T3) 6 days prior to treatment. 2 mg E2 per animal were administered orally once a day for 2 (2d) or 5 (5d) consecutive days. 4 µg rhTSH (a converted human equivalent dose) per animal was given intraperitoneally once a day on two consecutive days. Serum for T4 measurement was obtained by terminal retro-orbital bleed from anesthetized mice 2 h after the last dose. The total number of animals per treatment group is 4.
